# A Vision-Based System for In-Sleep Upper-Body and Head Pose Classification

**DOI:** 10.3390/s22052014

**Published:** 2022-03-04

**Authors:** Yan-Ying Li, Shoue-Jen Wang, Yi-Ping Hung

**Affiliations:** 1 Department of Computer Science and Information Engineering, National Taiwan University, Taipei 10167, Taiwan; hung@csie.ntu.edu.tw; 2 Tainan National University of the Arts, Tainan 72045, Taiwan; sjwang@tnnua.edu.tw

**Keywords:** sleep posture, sleep monitoring, head and upper-body detection, head and upper-body pose classification, deep multi-task learning

## Abstract

Sleep quality is known to have a considerable impact on human health. Recent research shows that head and body pose play a vital role in affecting sleep quality. This paper presents a deep multi-task learning network to perform head and upper-body detection and pose classification during sleep. The proposed system has two major advantages: first, it detects and predicts upper-body pose and head pose simultaneously during sleep, and second, it is a contact-free home security camera-based monitoring system that can work on remote subjects, as it uses images captured by a home security camera. In addition, a synopsis of sleep postures is provided for analysis and diagnosis of sleep patterns. Experimental results show that our multi-task model achieves an average of 92.5% accuracy on challenging datasets, yields the best performance compared to the other methods, and obtains 91.7% accuracy on the real-life overnight sleep data. The proposed system can be applied reliably to extensive public sleep data with various covering conditions and is robust to real-life overnight sleep data.

## 1. Introduction

Good sleep quality helps the mind and body remain healthy. Irregular sleeping patterns can increase the risk of diabetes, obesity, and cardiovascular disease [1,2]. Numerous studies have shown that sleep postures can serve as an indicator of sleep health. Monitoring in-bed postures provides valuable information regarding the intensity of dreams [3], risk of pressure ulcers [4], patients’ mobility [5], obstructive sleep apnea syndrome [6], risk of spinal symptoms [7], and quality of sleep [8]. Therefore, sleep behavior monitoring is a critical aspect of healthcare management. Moreover, home sleep testing is becoming critical at present owing to the overwhelmed healthcare system resulting from the COVID-19 pandemic [9]. A contact-free sleep monitoring system is necessary for healthcare in the non-contact era.

Traditionally, trunk posture has been used to study the impact on sleep, but a growing number of studies have indicated that head posture has a crucial impact on sleep as well. For example, head posture has a noticeable impact on obstructive sleep apnea (OSA) independent of trunk posture [10,11], and rotation of the head from the supine to lateral posture decreased the OSA severity significantly while the trunk was in the supine posture [10]. A recent study [12] found that head posture during sleep affects the clearance of neurotoxic proteins from the brain. In addition, head posture is related to sleep quality. Sleepers who spend more time on their backs with their heads straight have a high probability of experiencing poor sleep quality [13]. As whole-body shapes under blankets cannot be seen using popular home security cameras, only head and upper-body appearance are considered in this study. Monitoring in-bed head and body posture simultaneously can help us address these issues and better understand sleep behaviors.

Non-invasive technology, such as a computer vision-based system, is better suited to monitoring sleep posture with no disturbance and low cost. However, region detection is usually performed separately from pose classification, and the detecting region is traditionally set as the bed region instead of the human body. To make sleep monitoring practically useful, it needs efficient and robust head and body detection modules to localize heads and bodies with large variations in illumination and coverings for the pose classification tasks. Therefore, this paper presents a sleep monitoring system combining efficient body localization and pose classification using a home security camera. Our system is applicable to both RGB and infrared (IR) modes.

The number of sleep posture datasets with annotations is limited due to privacy and cost concerns. Moreover, the sleep streams are of very long duration, and it needs an efficient way to process the enormous amount of data. The multi-task learning method is applied to overcome the challenge of the sleep dataset. A unified framework is proposed to simultaneously detect and classify head and upper-body posture. All detection and classification tasks are trained and tested simultaneously in an all-in-one convolutional neural network (CNN). This decreases training times, reduces latency during inference, reduces storage space, and is easier to train, deploy, and maintain. The proposed method can save time and the amount paid for processing sleep videos.

To our knowledge, this is the first attempt to merge in-sleep head and upper-body detection as well as head and upper-body pose classification together in one unified network. In addition to recognizing sleep posture, synopses of sleep postures, sleep duration, and related sleep quality indicators are provided. This information can help medical professionals make suitable health recommendations. It can also help users better understand their sleep habits and find ways to improve and maintain sleep health.

The contributions of this work are as follows: (1) this is the first sleep posture study using a single infrared camera and a unified framework for simultaneously detecting and classifying upper-body and head poses during sleep, (2) an all-in-one CNN model with high accuracy is proposed to detect and classify sleep posture, which is robust to realistic sleep conditions, various covering conditions, and variable illumination, (3) a synopsis of sleep postures is provided for analysis and diagnosis of sleep behavior.

The remainder of this paper is organized as follows. Section 2 reviews related work of sleep pose classification and multi-task learning. Section 3 gives an overview of the proposed framework and explains the SleePose-FRCNN-Net architecture and SleePose-FRCNN-Net training strategy in detail. Section 4 describes the datasets used for training and testing and provides experimental results and comparisons with other methods. The discussion of this study is presented in Section 5. Finally, our work is concluded in Section 6.

## 2. Related Works

This work is related to sleep posture classification and multi-task learning. In this section, the literature is briefly reviewed regarding these two topics.

### 2.1. Sleep Posture Classification

Sleep posture classification is challenging owing to various reasons, such as variation in the viewing conditions while monitoring human sleep and lack of publicly available sleep datasets because of privacy and cost concerns. In addition, occlusion of the body under a blanket increases the difficulty of detecting and tracking human poses.

Several studies estimate the posture of bodies sleeping in beds using a high-cost pressure-sensing mat [14] or high-resolution thermal camera [15]. However, these devices are too expensive for home use.

Some studies use depth cameras [16,17,18,19]. Grimm et al. [16] use depth maps to detect sleep posture. However, their method does not distinguish between supine and prone postures. Klishkovskaia et al. [19] use Kinect v2 skeleton for the automatic classification of human posture detection. However, their method requires the subject to avoid any covering. In addition, when using depth cameras, the depth measurements may suffer from various noise factors such as ambient light, scene geometry, and glossy reflection [20,21]. The ambient light has an influence on the measurement and correlation of disparities. The scene geometry includes the distance to the object that impacts the error of depth measurement and the occluded or shadowed scenes that can lead to outliers in 3D reconstruction. The reflective surfaces can lead to noise in the depth measurement [21]. Thus, using depth cameras does not always guarantee high accuracy. In recent years, IR cameras have been commonly used for home surveillance. Taking into consideration deployment and prevalence factors, a sleep monitoring system using a single IR camera was chosen to design.

Few studies have focused on head and upper-body pose classification during sleep in comparison with full-body pose classification. Choe et al. [22] employ motion analysis to determine sleep and wake states using a general-purpose head detector. An accuracy of 50% is achieved overnight. A CNN model for tracking upper body joints in the clinical environments from RGB-video is proposed in [23]. The system focuses on monitoring the patient’s pose for clinical studies. Liu and Ostadabbas [24] propose a pre-trained CNN called convolutional pose machine for in-bed pose estimation using a near-infrared modality. Their data is obtained from mannequins in a simulated hospital room. In another approach, a CNN algorithm is developed to detect upper-body and head posture with blankets during sleep [25]. It focuses on the classification of posture without detecting the upper-body and head region. 

Recently, benefited from computer vision technologies, an increased number of research efforts have advanced camera-based sleep posture monitoring. A recent deep learning method distinguishes obstructive sleep apnea and central sleep apnea by tracking body movements using an IR camera [26]. Lyu et al. [27] use an object detection algorithm and human pose estimation algorithm to classify sleep posture without covering. A domain adaption-based training strategy [28] is proposed to estimate in-bed human poses using RGB and thermal images. Another study [29] presents a non-contact sleep monitoring system through a transfer learning strategy. Their study mainly focuses on trunk posture instead of head posture.

Table 1 compares the proposed method and previous methods for sleep posture classification. To the best of our knowledge, no previous work has integrated head detection, upper-body detection, head pose classification, and upper-body pose classification into one sleep monitoring system. 

### 2.2. Multi-Task Learning (MTL)

MTL is an excellent solution to share common knowledge among multiple related tasks. Learning correlated tasks jointly can improve performance and offers good generalization ability compared with single-task learning [30].

In a previous study [31], a heterogeneous multi-task model is trained for human pose estimation. They show that the regression network benefits considerably from the various body-part detection tasks. In another study [32], an accurate and cost-efficient MTL framework is employed for simultaneous face detection and 3D head pose estimation.

Research on applying MTL to sleep posture classification is limited. Piriyajitakonkij et al. [33] propose a multi-task learning network to classify sleep postural transition and sleep turning transition. Their application is based on the Ultra-Wideband radar system.

To our knowledge, this is the first attempt to merge in-sleep head and upper-body detection as well as head and upper-body pose classification in a single network.

## 3. Materials and Methods

**Figure 1:**
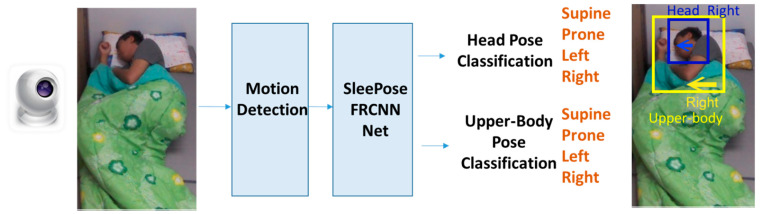
Architecture of the proposed framework.

This study designs and implements a non-contact sleep monitoring system to perform head and upper-body detection and pose classification. Figure 1 shows an overview of our framework, which consists of two modules: 

*3.1. Motion detection*: A motion detection algorithm is applied to trigger sleep video processing.

*3.2. SleePose-FRCNN-Net*: Head and upper-body detection and pose classification: a deep multi-task learning (DMTL) network for head and upper-body detection and pose classification (supine, prone, left, or right).

### 3.1. Motion Detection

A motion detection algorithm called Visual Background Extractor (ViBe) [34] is applied to reduce video processing. The ViBe is a rapid background modeling technique for video sequences. The method is robust and efficient for natural background scenes. The basic idea of the algorithm is to collect background samples for each pixel point to build background models. The elements in the background samples are selected randomly from the pixels around the pixel and used to update the background model. ViBe algorithm mainly consists of three aspects: the classification process of pixels, the initialization of the background mode, and the update strategy of the background model [34]. 

#### 3.1.1. The Classification Process of Pixels

A background model is built of N background samples for each pixel of the video frame. Equation (1) represents the background model:

(1) $$M\left(x\right)=\left\{v_{1},\;v_{2,\;\;}\ldots ,v_{N}\right\}\;,$$

where N is the number of background sample sets and v(x) is the value of pixel *x* in the image. The Euclidean distance between v(x) and the pixel of model samples M(x) is used to determine whether a pixel is similar to the background model samples. If the distance is less than threshold *R*, the count of the set intersection of the pixel and model samples increases. According to Equation (2), if the count of set intersection is larger than or equal to a given threshold ${\#}_{min}$, the pixel is classified as background; otherwise, it is classified as foreground.

(2) $$\;\;\;\#\left\{S_{R}\left(v\left(x\right)\right)\cap \left\{v_{1},\;v_{2,\;\;}\ldots ,v_{N}\right\}\right\}\geq {\#}_{min}\;x\;\in \mathrm{background}\;\#\left\{S_{R}\left(v\left(x\right)\right)\cap \left\{v_{1},\;v_{2,\;\;}\ldots ,v_{N}\right\}\right\}<{\#}_{min}\;x\;\in \mathrm{foreground}$$

where $S_{R}\left(v\left(x\right)\right)$ is a circle centered on *v*(*x*) and has radius *R*.

#### 3.1.2. The Initialization of the Background Mode

The first video frame is used to initialize the background model. Certain pixels are randomly selected from their neighborhood to fill the background sample set for each pixel.

#### 3.1.3. The Update Strategy of the Background Mode

The update strategy of the ViBe algorithm is the random replacement. A pixel classified as the background has 1 chance in 16 of being selected to update its background sample set with its current pixel value. This method covers a large time window and better detects the slow-moving targets.

#### 3.1.4. Pre- and Post-Processing

Preprocessing includes histogram equalization for adjusting image intensities across varying bedroom lighting. After applying the ViBe algorithm, the segmentation mask that includes moving object pixels in the frame is obtained. The erosion and dilation processes are used to process the segmentation mask to improve accuracy. The operation is applied to remove foreground blobs whose area is smaller and fill holes to keep the completion of objects. 

### 3.2. SleePose-FRCNN-NET—Head and Upper-Body Detection and Pose Classification

The network aims to recognize the head and upper-body poses during sleep from an image. To this end, the SleePose-FRCNN-Net, a DMTL network, is introduced to jointly detect and classify head pose and upper-body pose from the input image. The Faster R-CNN [35] is adopted as a basic detection framework in our work. The main reason for choosing the Faster R-CNN framework to detect sleep postures is that it has higher accuracy than other deep learning-based detectors [36]. The head detection, upper-body detection, head pose classification, and upper-body pose classification are combined in a unified convolutional neural network.

#### 3.2.1. SleePose-FRCNN-NET Architecture 

**Figure 2:**
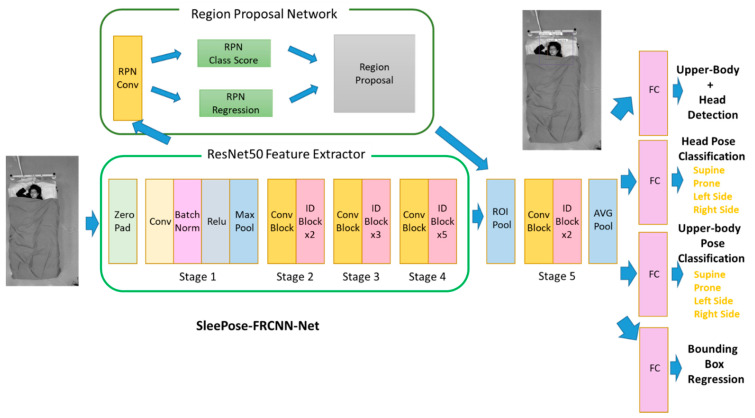
SleePose-FRCNN-Net architecture.

As shown in Figure 2, the framework consists of four major modules: feature extraction module, region proposal network (RPN) module, head and upper-body detection module, and head and upper-body pose classification module.

1. Feature Extraction Module

From a given image, convolutional features are extracted first. The ResNet-50 [37] network is employed as the backbone architecture in the feature extraction module and initializes the network using the weights pre-trained on the ImageNet dataset. The ResNet-50 model consists of five stages, each with a residual block. Each residual block consists of a set of repeated layers. The Faster R-CNN model allows different input image sizes in the RGB channels. The shortest side can be any number larger than 600 pixels, and the longest side smaller than 1000 pixels, as suggested by the Faster R-CNN paper [35].

2. RPN Module

The RPN in Faster R-CNN [35] is developed for proposing candidate regions. The RPN predicts a set of candidate object proposals and corresponding objectness scores from feature maps. It is built on top of the res4f layer of the ResNet-50 network, followed by an intermediate 3 × 3 convolutional layer with 512 channels and two sibling 1 × 1 convolutional layers for classification and bounding box regression. The anchor-based method [38] is proposed to detect objects with multiple scales and aspect ratios. This method generates nine bounding boxes, which includes three scales ($128^{2}$, $256^{2}$, and $512^{2}$ pixels) and three aspect ratios (1:1, 1:2, and 2:1). Finally, the RPN classifies each bounding box’s category (an object or not) and regresses four coordinates. The non-maximum suppression (NMS) algorithm prunes redundant, overlapping bounding boxes at the finetuning step.

3. Head and Upper-Body Detection Module

With the predicted bounding boxes generated by RPN, the RoI pooling layer [38] is adopted to extract feature maps from regions. The feature map passes through a residual block and an average pooling layer. Finally, a fully connected layer and softmax are added to classify the results into three classes (head, upper-body, and background) and output bounding box regression.

4. Head and Upper-Body Pose Classification Module

The head and upper-body pose class can be considered as a subcategory in head and upper-body detection. Two fully connected layers are appended with four outputs (supine, prone, left side, right side). Each of the fully connected layers makes predictions for individual tasks.

#### 3.2.2. SleePose-FRCNN-NET Training

The training DMTL network contains the ResNet50 backbone network and sub-networks. The multi-task loss function is used to train head and upper-body detection, head and upper-body pose classification, and bounding box regression. Here, the loss functions for each task are discussed.

1. Head and Upper-Body Detection

This detection performs head and upper-body classification and uses categorical cross-entropy loss. The loss function is defined as the following Equation (3).

(3) $$L_{class}=-\sum_{i=1}^{k}y_{i}\log\left({\hat{y}}_{i}\right),$$

where $y_{i}$ is the ground truth and$\;\hat{y}$ is the predicted score for each class i.

2. Head Pose Classification

Predicting the head pose is a multiple-class problem. The categorical cross-entropy is used as a loss function. The loss is considered only if the box is detected as a head. The loss function is defined as the following Equation (4).

(4) $$L_{head}=-\sum_{i=1}^{k}y_{i}\log\left({\hat{y}}_{i}\right),$$

where $y_{i}$ is the ground truth pose class and$\;\hat{y}$ is the predicted score for the pose class.

3. Upper-Body Pose Classification

Predicting the upper-body pose is a multiple-class problem. The categorical cross-entropy is used as a loss function. The loss is considered only if the box is detected as the upper body. The loss function is defined as the following Equation (5).

(5) $$L_{body}=-\sum_{i=1}^{k}y_{i}\log\left({\hat{y}}_{i}\right),$$

where $y_{i}$ is the ground truth and$\;\hat{y}$ is the predicted score for each pose class i.

4. Bounding Box Regression

The bounding box regression is used to tighten the bounding boxes for each identified region. For bounding box regression, the smooth L1 loss is used. The loss function is defined as the following Equation (6).

(6) $$L_{box}=\sum_{i\epsilon \left\{x,y,w,h\right\}}^{k}L_{1}^{smooth}\left(t_{i}^{k}-{\hat{t}}_{i}\right),$$

where $t^{k}=\left(t_{x}^{k},t_{y}^{k},t_{w}^{k},t_{h}^{k}\right)$ denotes four predicted parameterized coordinates for class k and ${\hat{t}}_{i}$ indicates ground truth coordinates.

5. Overall Multi-Task Loss

The overall loss function is the weighted average of all the losses defined above. The overall loss function is defined as the following Equation (7).

(7) $$L=\lambda_{class}L_{class}+\lambda_{head}L_{head}+\lambda_{body}L_{body}+\lambda_{box}L_{box,}$$

where $\lambda_{i}$, i∈{class, head, body, box} are loss weights to balance their contributions to the overall loss.

6. Parameter Setting

The model was trained on an Nvidia GeForce RTX 2070 GPU using the Adam optimizer with a learning rate of 0.00001. All the tasks were trained end-to-end, with a validation-based early stopping, in order to avoid overfitting. The development environment is built on the Keras framework.

### 3.3. Data Augmentation

Data augmentation is used to expand the training dataset to reduce overfitting and improves generalization. For this study, data augmentation was performed when training the live streaming dataset, in the form of applying randomly rotating input images between 0 and 15 degrees, and randomly adjusting contrast and brightness of images.

### 3.4. Sleep Analysis—Posture Focused

Our system provides a pictorial representation of sleep postures and posture-related indicators that have been proven to be highly associated with sleep quality [39]. The indicators include (a) shifts in sleep posture, (b) the number of postures that last longer than 15 min, (c) average duration in postures, and (d) sleep efficiency (based on turning) [39]. Table 2 gives detailed descriptions of indicators of sleep quality.

Sleep postures and movements are associated with sleep quality. Our system classifies head and upper-body sleep postures into four clinical standard categories: supine, left, right, and prone. 

Several studies reveal that the number of shifts in sleep posture, postures that last longer than 15 min, and nocturnal movements are related to lifestyle and insomnia symptoms. Generally, healthy people shift their posture between 10 and 30 times per night [40]. However, too much tossing and turning during the night indicates poor sleep quality. The duration in a particular posture has been associated with various medical conditions. Patients remaining in the same posture for long periods have an increased risk of pressure injury. Several clinical guidelines recommend that patients change posture at least every two hours [41].

Here, the synopsis of sleep posture and movement that can be assessed for further sleep measurement and study is provided.

## 4. Experimental Results and Analysis

### 4.1. Datasets 

Several datasets were used to train and test our CNN model. The Simultaneously-Collected Multimodal Lying Pose (SLP) dataset [42] with annotated head and upper-body position was used for training and testing. The imLab@NTU Sleep Posture Dataset (iSP) dataset [43] was used, and a YouTube dataset was created for training and testing. 

#### 4.1.1. Simultaneously-Collected Multimodal Lying Pose (SLP) 

Simultaneously-collected multimodal Lying Pose (SLP) is a large-scale in-bed pose dataset. The SLP dataset contains images from RGB and thermal cameras. RGB images were used for our study. 

**Figure 3:**
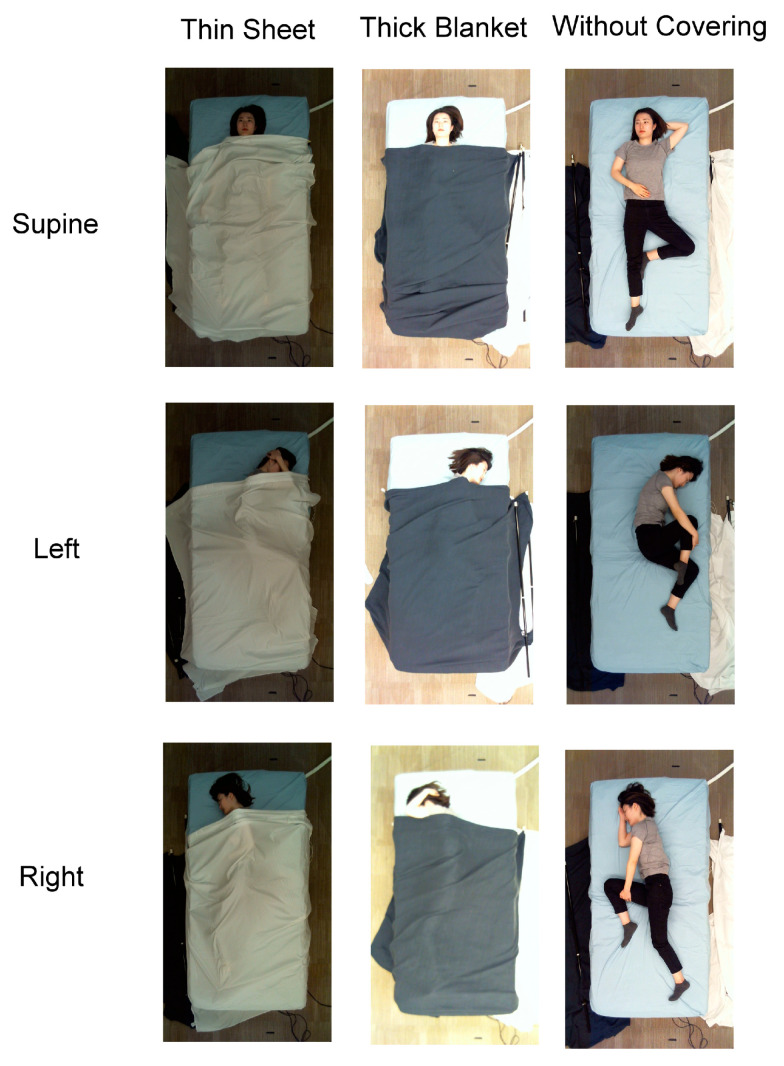
Sample images for each pose class from the SLP database [42].

Images from 109 subjects were collected under three main categories: supine, left side, and right side. Example images of the SLP dataset are shown in Figure 3. The data from 102 subjects was collected in the living room and seven subjects in the hospital room. The samples were collected under various cover conditions: no covering, a thin sheet, and a thick blanket. Table 3 describes the details of the SLP dataset.

#### 4.1.2. imLab@NTU Sleep Posture Dataset (iSP)

1. Pilot Experiment

**Figure 4:**
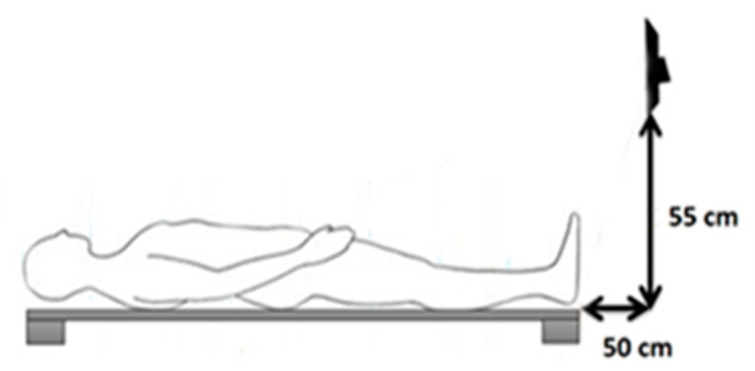
Illustration of the system in the experiment [43].

An experimental environment was set up for recording sleep postures. Microsoft Kinect was mounted at a horizontal distance of 50 cm and vertical distance of 55 cm above the bed, so that its field of view can cover the bed and entire body of a subject (Figure 4).

**Figure 5:**
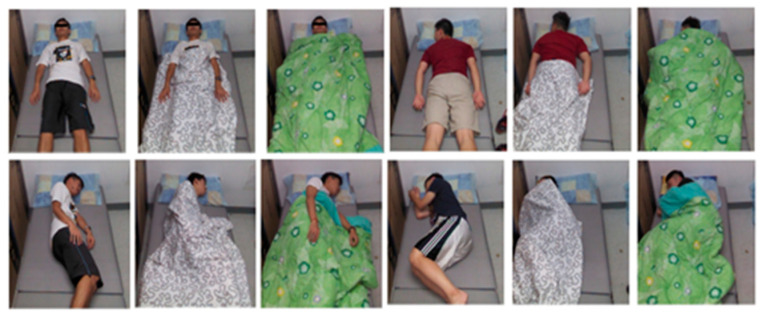
Examples of images from the iSP pilot dataset [43].

**Figure 6:**
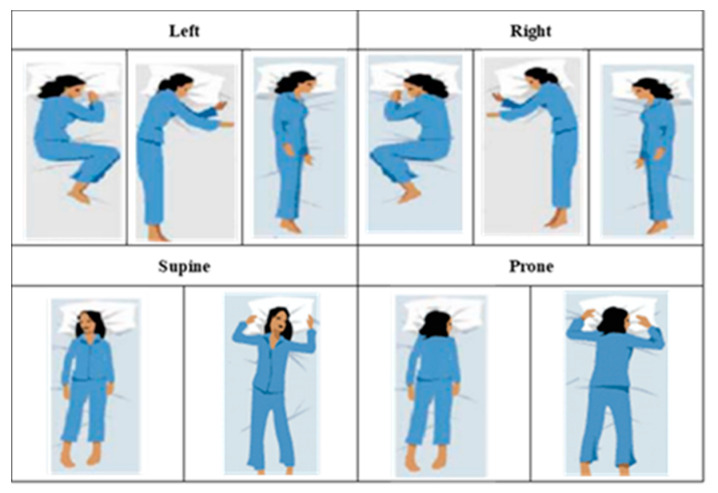
The 10 types of sleep postures [43].

RGB and depth images were collected simultaneously. Only RGB images were used in this work, as shown in Figure 5. Thirty-six subjects participated in the experiment, of which 29 subjects were male and seven were female. Based on Sleep Assessment and Advisory Service (SAAS)’s research, 10 types of sleep postures were chosen, including the left side in fetal posture, left side in yearner posture, left side in log posture (LEFT set), right side in fetal posture, right side in yearner posture, right side in log posture (RIGHT set), supine in hand-up posture, supine in hand-down posture (SUPINE set), prone in hand-up posture, and prone in hand-down posture (PRONE set) as shown in Figure 6. The total time of the experiment of each subject was 10 min, and each subject mimicked each type of sleep posture lasting 1 min via manual guidelines. Sleep postures were collected under three cover conditions: no covering, a thin sheet, and a thick blanket. Table 4 describes the details of the iSP dataset.

2. Real-Life Sleep Experiment

**Figure 7:**
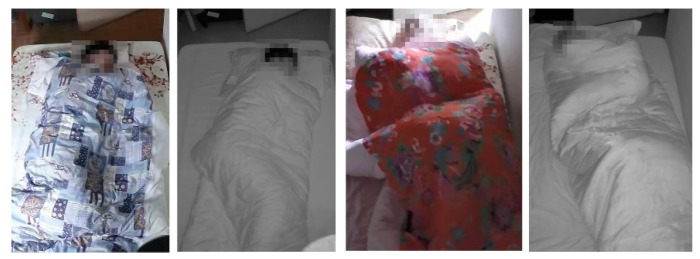
Examples of RGB/IR images from the iSP real-life dataset.

Four healthy adult subjects participated in the real-life study. The video was recorded at 20 frames per second from a home security camera during each subject’s sleep. The duration of each subject’s record and the number of frames used for evaluation are shown in Table 5. This dataset includes RGB images in day mode and IR images in night mode, as Figure 7 shows. A home security day-and-night camera was fit onto a custom-built mount that stood about 200 cm tall to the front of the bed. To increase posture variance and diversity, image frames were manually labeled and selected from minor movement and non-movement periods.

#### 4.1.3. YouTube Dataset 

With the growing popularity of video-sharing sites such as YouTube, many people continuously broadcast daily events in their life, including while sleeping. Four sleep streams were collected from YouTube, whose duration varied from 5 to 8 h. The dataset included RGB and IR images. It contained sleep data with various poses, coverings, and illumination. A sampling rate of one frame per second was used for our experiments and the redundant frames were discarded. The time of each sleep stream and the number of frames are presented in Table 6.

### 4.2. Evaluation on SLP Dataset 

The model was trained on 4050 samples of the first 90 subjects recorded in the room and validated on 540 samples of the remaining 12 subjects. The model was tested on 315 samples recorded in the hospital. As the SLP dataset is not annotated for head and upper-body detection, ground truth labels for head and upper-body regions were annotated manually.

The mean average precision (mAP) result of head and upper-body detection is presented in Table 7. As can be seen from the table, our method is more robust to environmental changes than the YOLOv3 [44] and YOLOv4 [45] methods. Our approach applies whether the image is captured in the room or the hospital.

**Figure 8:**
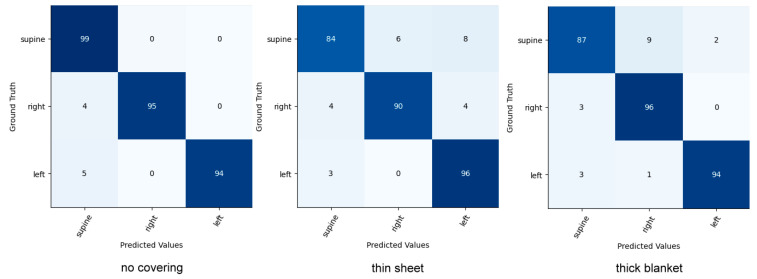
Confusion matrix for head pose classification on the SLP dataset.

**Figure 9:**
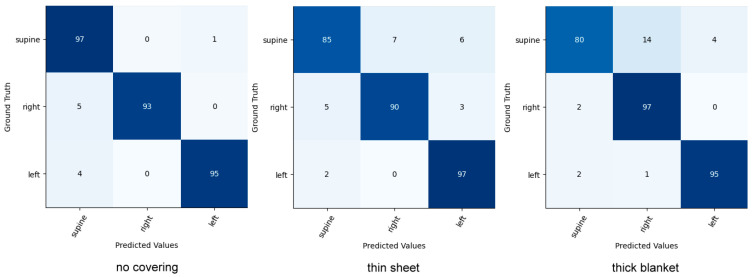
Confusion matrix for upper-body pose classification on the SLP dataset.

For head and upper-body pose classification, our method was compared with Akbarian et al.’s work [25], Mohammadi et al.’s work [46], and the Inception network [47], which was used to extract deep features in the study of Torres et al. [48] for the classification of sleep poses. Given that the above methods cannot detect heads and upper bodies, detections from the SleePose-FRCNN-Net are provided. Table 8 and Table 9 present the results of our approach and the other methods on the SLP dataset. As can be seen, our method achieves the highest accuracy. Confusion matrices of our models are shown in Figure 8 and Figure 9 for head and upper-body poses, respectively.

### 4.3. Evaluation on the iSP Dataset

#### 4.3.1. iSP Pilot Dataset

Next, the model was trained and tested on the iSP pilot dataset. In this experiment, 15,000 samples from 25 subjects were used to train the model, and the remaining 6600 samples from 11 subjects were used to test the model. The model was trained and tested with three covering conditions. The detection results in terms of mAP are presented in Table 10. The average processing time of SleePose-FRCNN-Net is about 0.7 s. Although the processing time by our method is longer than YOLOv3 (0.43 s), our accuracy is higher than YOLOv3′s accuracy, as shown in Table 7 and Table 10.

1. Comparison with Posture Classification

**Figure 10:**
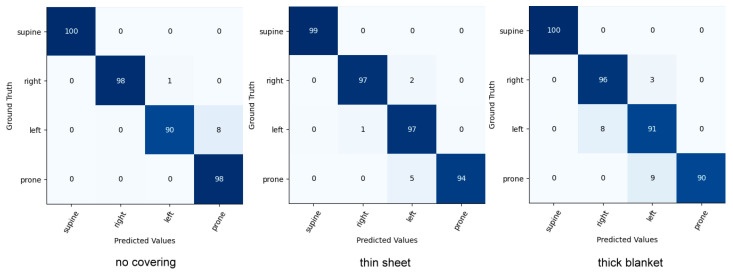
Confusion matrix for head pose classification on the iSP pilot dataset.

**Figure 11:**
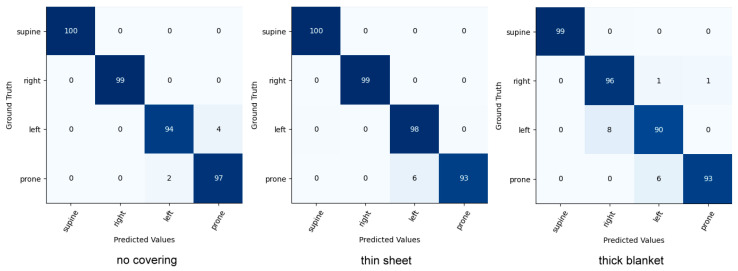
Confusion matrix for upper-body pose classification on the iSP pilot dataset.

Head and upper-body classifications were evaluated on the iSP pilot dataset. Our model was compared to the current state-of-the-art sleep posture classification model. The results can be seen in Table 11 and Table 12. The results show that our model achieves higher accuracy in three cover conditions. As illustrated in the confusion matrix in Figure 10 and Figure 11, incorrect predictions occur rarely.

2. Comparison with General Human Pose Estimation

**Figure 12:**
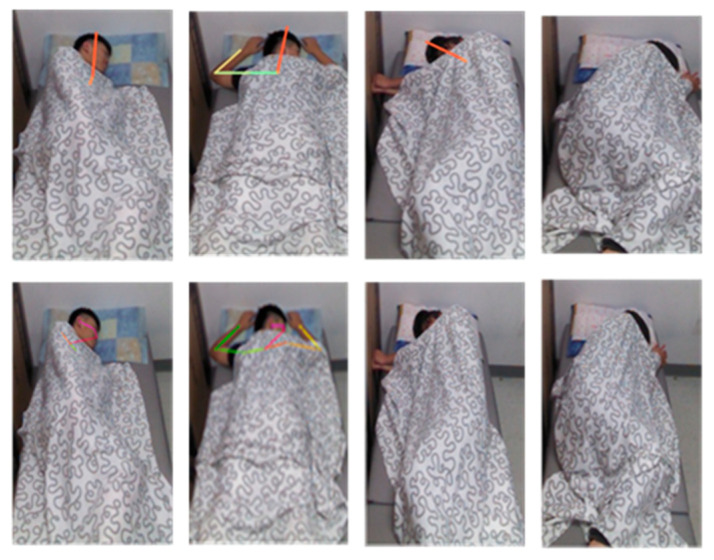
Repurposed SimpleBaseline [49] (first row) and OpenPose [50] (second row) performance on the iSP dataset.

Pose estimation results were compared with SimpleBaseline [49] and OpenPose [50], which is the most classical pose estimation model and is used to recognize the types of sleep postures in the study of Lyu et al. [24]. Although these models have satisfactory performance in the general case, they do not perform well in the iSP dataset. This is likely because some body parts of an individual are occluded by a blanket. For example, Figure 12 illustrates that SimpleBaseline and OpenPose do not detect the individual’s body parts under the covering.

#### 4.3.2. Real-Life Sleep Experiment

Head and upper-body classifications were evaluated on the iSP real-life dataset. Training and testing sets were chosen from different fragments of sleep. The data ratio is 80% and 20% for training and testing sets, respectively. After training, the model obtains an overall classification accuracy of 91.67% for detecting the upper-body sleep postures and 94.37% for detecting the head sleep postures. 

**Figure 13:**
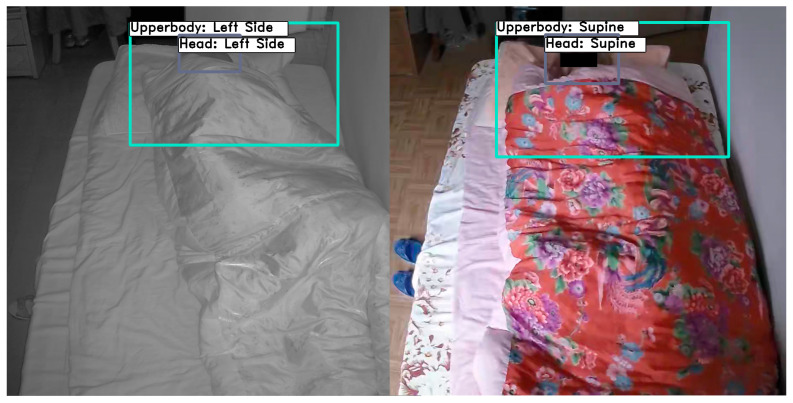
Sleep posture detection on real-life data. The left column shows images in IR mode and the right column shows in RGB mode.

Experimental results demonstrate that the proposed model is applicable to the real environment. Our model can handle sleep posture detection and classification with RGB and IR images, as shown in Figure 13.

### 4.4. Evaluation on YouTube Dataset

The individual model was trained using the data from each subject. Training and testing sets were chosen from different fragments of sleep. The data ratio is 80% and 20% for training and testing sets. The detection results in terms of mAP are presented in Table 13. The averaged accuracy of each subject is presented in Table 14 and Table 15. Across four subjects, our method achieves an overall classification accuracy of 96.92% for detecting the four main sleep postures: supine, prone, right, and left. 

**Figure 14:**
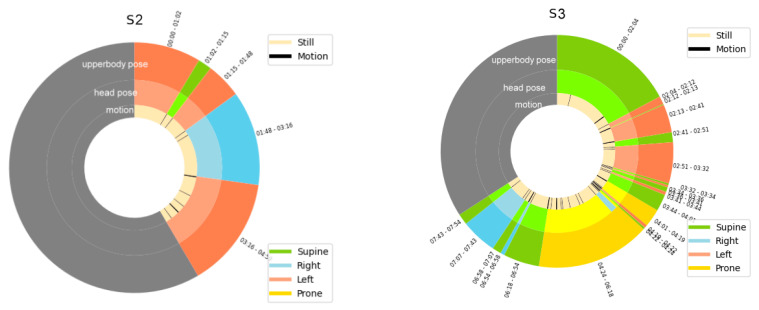
Pictorial representation of sleep poses and motion over time of subjects.

The ability of our system to detect both sleep posture and movement is exhibited with a pictorial representation, as shown in Figure 14. The circular diagram shows the state of 12 h. It is divided into segments to represent the duration of different sleep poses. A grey area indicates a waking state. The diagram shows that subject S2 had few body movements, few shifts in sleep posture, high sleep efficiency, and few awakenings.

Our system provides posture-related indicators of sleep quality, as presented in Table 16. According to research, a higher percentage of time in bed with fewer shifts indicates more efficient sleep.

### 4.5. Computational Performance

The average processing time per image by our method is about 0.7 s. The experiment was performed on the desktop computer explained in Section 3.2.2. In addition, our algorithm was implemented in python using the Keras mechanism. Python is a slow programming language because of its dynamically typed feature and limited to using a single processor during its application. Therefore, if our algorithm is implemented in the C language in the future, the processing time will be significantly reduced with the same accuracy.

## 5. Discussion

The paper presents a new approach to sleep posture monitoring based on a deep multi-task learning network. The innovation of this work is integrating head detection, upper-body detection, head pose classification, and upper-body pose classification into one sleep monitoring system. The upper-body pose and head pose information during sleep are obtained simultaneously. The proposed method was evaluated using laboratory-based simulation datasets such as the SLP, iSP, and real-life datasets such as YouTube, and both achieved impressive results. The work demonstrates practical value because the system uses a single 2D IR video camera and applies to various covering conditions and variable illumination.

Most of the existing techniques for sleep posture monitoring only focus on posture classification methods; however, the proposed method combines head detection, upper-body detection, head pose classification, and upper-body pose classification into one united framework. The proposed method is more robust and accurate than the methods used in other papers [25,46,47], which use a deep learning classification model for sleep monitoring. The proposed system takes a video feed from an IR camera and analyzes the video stream. The ability of the proposed approach to accurately detect both upper-body and head postures provides valuable information for sleep studies.

Although our method was evaluated on adults, the proposed method can be applied to babies and children using transfer learning based on a pre-trained network. The model trained on the adult data is initialed, then finetunes that model on the infant and children dataset.

To analyze the generalization ability of the proposed model, the model was trained on the SLP training dataset and tested on the iSP pilot testing dataset. The model obtains an average classification accuracy of 89.17% for classifying the upper-body sleep postures and 91.25% for classifying the head sleep postures. Table 17 shows that our model generalizes well on previously unseen, new data. In addition, the camera placement and monitoring zone have a significant impact on the overall performance of video analysis. For posture detection and classification, to increase confidence in the system, it is recommended that a camera is installed at the 2–2.5 m height. The whole body can be covered in the view. It is necessary to avoid the extreme angle view (from the side).

This research, however, is subject to several limitations. Due to lack of manpower and labeled sleep posture data, a small amount of data in the real-life dataset was used in this study. A large amount of labeled data is needed to adapt to challenging environments, such as variations in human appearance and arbitrary camera viewpoints. This limitation could be mitigated with a semi-automatic annotation tool in the future.

## 6. Conclusions

This paper presented a non-contact video-based framework for simultaneous head and upper-body detection and pose classification during sleep. All detections and classifications were trained and tested simultaneously in a single multi-task network. Experimental investigations on three available datasets show that the proposed system can be applied reliably to extensive public sleep data with various covering conditions and is robust to real-life overnight sleep data. The real-life application achieves a high accuracy of 91.7% in upper-body pose classification. Furthermore, a sleep posture and movement synopsis is provided to assess sleep quality and irregular sleeping habits.

## Figures and Tables

**Table 1:** Comparison of previous studies and the proposed method on sleep posture classification.

Sensor	Method	Dataset Used	Advantages	Limitations
Pressure-sensing mat	3D human pose estimation based on deep learning method [14]	Simulation dataset	A pressure-sensing mat is robust to covering.	A pressure-sensing mat has high cost and complex maintenance for home use.
Thermal camera	Human pose estimation based on deep learning method [15]	Simulation dataset	A thermal camera is robust to illuminationchanges and covering.	A thermal camera has high cost for home use.
Depth camera	Sleep posture classification based on deep learning method [16,18]	Simulation dataset	A depth camera is robust to low light intensity.	- Depth measurements of depth cameras suffer from various noise factors. - The depth camera is not prevalent at home surveillance.
Infrared camera	Sleep vs. wake states detection in young children based on motion analysis [22]	Real sleep data	- A single infrared camera is convenient and low-cost. - A general-purpose head detector is used.	This method only succeeds for 50% of nights.
	Human pose estimation based on deep learning method (OpenPose) [24,27]	Simulation dataset	The method can extract features of the skeleton effectively.	Their data [24] is obtained from mannequins in a simulated hospital room, and this method cannot perform well on real data [25].
	Sleep posture classificationbased on deep learning method [25,29]	Simulation dataset	The deep learning method can achieve good accuracy.	It focuses on classifying posture without detecting the upper-body and head region.
	Sleep posture detection and classification based on deep learning method (proposed method)	Simulation and real sleep dataset	A unified framework for simultaneously detecting and classifying upper-body pose and head pose is proposed.	Training personal data to learn CNN is required.

**Table 2:** Indicators of sleep quality.

Indicator	Description	Unit
Shifts in sleep posture	Count of posture changes	Count
Number of postures that last longer than 15 min	Count of postures with duration longer than 15 min	Count
Average duration in a posture	Mean of the posture duration	Minutes
Sleep efficiency	Percentage of time without turning	Percentage

**Table 3:** The details of the SLP dataset.

Dataset	Sleep Postures Categories	Covering Condition	Set	Recorded Environment	Number of Subjects	Number of Images per Covering Condition
SLP [42]	Supine, left side, and right side	No covering, thin sheet, and thick blanket	Train	Living room	90	4050
			Validation	Living room	12	540
			Test	Hospital room	7	315

**Table 4:** The details of the iSP pilot dataset.

Dataset	Sleep Postures Categories	Covering Condition	Set	Recorded Environment	Number of Subjects	Number of Images per Covering Condition
iSP [43]	Supine, left side, right side, and prone	No covering, thin sheet, and thick blanket	Train	Lab	25	15,000
			Test	Lab	11	6600

**Table 5:** The iSP real-life dataset summary.

Subject	Hours	Number of Frames
S1	8	576,000
S2	6	432,000
S3	7	504,000
S4	8	576,000

**Table 6:** YouTube dataset summary.

Subject	Hours	Number of Frames
S1	8	28,800
S2	5	18,800
S3—Day 1	8	28,800
S3—Day 2	7.5	27,000

**Table 7:** Results of detection on the SLP dataset.

Model	mAP (No Covering)		mAP (Thin Sheet)		mAP (Thick Blanket)	
	Val	Test	Val	Test	Val	Test
YOLOv3 [44]	99.26	96.84	99.58	69.30	99.70	64.34
YOLOv4 [45]	98.67	95.59	99.54	91.27	99.29	83.84
SleePose-FRCNN-Net	99.82	99.86	99.97	99.91	99.64	92.31

**Table 8:** Results of head pose classification on the SLP dataset.

Model	Accuracy (No Covering)		Accuracy (Thin Sheet)		Accuracy (Thick Blanket)	
	Val	Test	Val	Test	Val	Test
Akbarian [25]	95.61	87.50	96.70	83.66	97.00	85.52
Mohammadi [46]	85.56	78.47	79.42	71.90	85.19	71.03
Inception [47]	85.77	78.47	79.61	72.22	88.20	70.69
SleePose-FRCNN-Net	99.25	96.15	99.07	90.48	98.70	92.70

**Table 9:** Results of upper-body pose classification on the SLP dataset.

Model	Accuracy (No Covering)		Accuracy (Thin Sheet)		Accuracy (Thick Blanket)	
	Val	Test	Val	Test	Val	Test
Akbarian [25]	98.27	85.06	96.29	88.56	96.19	88.64
Mohammadi [46]	85.36	77.60	91.84	73.20	88.57	68.18
Inception [47]	86.51	66.12	89.98	82.20	88.57	64.29
SleePose-FRCNN-Net	99.44	95.24	99.07	91.11	98.70	91.11

**Table 10:** Results of detection on the iSP pilot dataset.

Model	mAP (No Covering)	mAP (Thin Sheet)	mAP (Thick Blanket)
YOLOv3 [44]	97.11	93.63	95.38
YOLOv4 [45]	99.61	94.11	98.59
SleePose-FRCNN-Net	99.82	94.67	99.64

**Table 11:** Results of head pose classification on the iSP pilot dataset.

Model	Accuracy (No Covering)	Accuracy (Thin Sheet)	Accuracy (Thick Blanket)
Akbarian [25]	87.62	92.10	91.80
Mohammadi [46]	77.90	74.82	71.05
Inception [47]	83.95	84.16	78.61
SleePose-FRCNN-Net	96.65	97.33	94.58

**Table 12:** Results of upper-body pose classification on the iSP pilot dataset.

Model	Accuracy (No Covering)	Accuracy (Thin Sheet)	Accuracy (Thick Blanket)
Akbarian [25]	87.62	92.10	91.80
Mohammadi [46]	77.90	74.82	71.05
Inception [47]	83.95	84.16	78.61
SleePose-FRCNN-Net	97.79	98.33	94.72

**Table 13:** Results of detection on the YouTube dataset.

Method	mAP			
	S1	S2	S3—Day 1	S3—Day 2
SleePose-FRCNN-Net	96.33	99.57	99.66	96.30

**Table 14:** Results of head pose classification on the YouTube dataset.

Method	Accuracy			
	S1	S2	S3—Day 1	S3—Day 2
SleePose-FRCNN-Net	89.87	90.67	95.44	92.38

**Table 15:** Results of upper-body pose classification on the YouTube dataset.

Method	Accuracy			
	S1	S2	S3—Day 1	S3—Day 2
SleePose-FRCNN-Net	98.21	96.10	97.62	98.25

**Table 16:** Sleep indicators of subjects.

	Subject			
	S1	S2	S3—Day1	S3—Day2
Shifts in sleep posture (n/hour)	1.63	1.00	2.38	2.53
Number of postures that last longer than 15 min (n/hour)	1.38	1.20	1.50	1.75
Average duration in a posture (min)	19.20	60.00	25.26	23.68
Sleep efficiency (percentage)	89.38	96.00	91.67	83.56

**Table 17:** Results of the proposed model that was trained on the SLP dataset and tested on the iSP pilot dataset.

Pose	Accuracy (No Covering)	Accuracy (Thin Sheet)	Accuracy (Thick Blanket)
Head	92.57	94.74	86.44
Upper-Body	92.92	91.10	83.51
